# Accurate size-based protein localization from cryo-ET tomograms

**DOI:** 10.1016/j.yjsbx.2024.100104

**Published:** 2024-06-26

**Authors:** Weisheng Jin, Ye Zhou, Alberto Bartesaghi

**Affiliations:** a Department of Computer Science, Duke University, Durham, USA; b Department of Biochemistry, Duke University School of Medicine, Durham, USA; c Department of Electrical and Computer Engineering, Pratt School of Engineering, Duke University, Durham, USA

**Keywords:** Cryo-electron tomography, 3D particle picking, Size-based object detection, Sub-tomogram averaging

## Abstract

Cryo-electron tomography (cryo-ET) combined with sub-tomogram averaging (STA) allows the determination of protein structures imaged within the native context of the cell at near-atomic resolution. Particle picking is an essential step in the cryo-ET/STA image analysis pipeline that consists in locating the position of proteins within crowded cellular tomograms so that they can be aligned and averaged in 3D to improve resolution. While extensive work in 2D particle picking has been done in the context of single-particle cryo-EM, comparatively fewer strategies have been proposed to pick particles from 3D tomograms, in part due to the challenges associated with working with noisy 3D volumes affected by the missing wedge. While strategies based on 3D template-matching and deep learning are commonly used, these methods are computationally expensive and require either an external template or manual labelling which can bias the results and limit their applicability. Here, we propose a size-based method to pick particles from tomograms that is fast, accurate, and does not require external templates or user provided labels. We compare the performance of our approach against a commonly used algorithm based on deep learning, crYOLO, and show that our method: i) has higher detection accuracy, ii) does not require user input for labeling or time-consuming training, and iii) runs efficiently on non-specialized CPU hardware. We demonstrate the effectiveness of our approach by automatically detecting particles from tomograms representing different types of samples and using these particles to determine the high-resolution structures of ribosomes imaged *in vitro* and *in situ*.

## Highlights

• Size-based particle picking algorithm efficiently locates proteins within tomograms.
• Does not require external templates, labeled data for training or access to GPUs.
• Uses contamination mask to reduce the number of false-positives and improve accuracy.
• Is faster and achieves higher accuracy than fully-supervised deep learning methods.
• Validated on *in vitro* and *in situ* datasets and on complexes with 300 kDa to 3 MDa sizes.

## Graphical abstract

**Figure:**
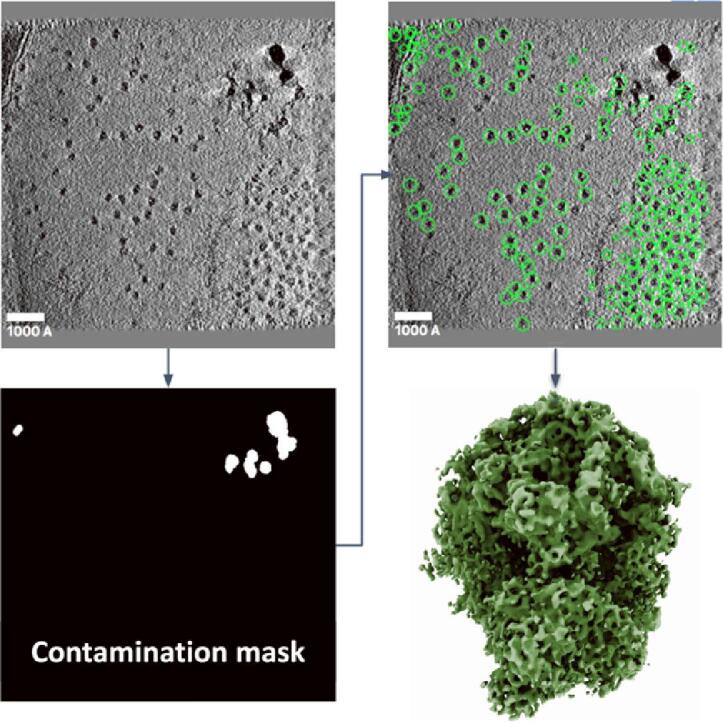

## 1 Introduction

Cryo-electron microscopy (cryo-EM) is an effective technique to image monodisperse protein samples in their frozen-hydrated state. By averaging the signal from multiple copies of the target of interest, single-particle analysis (SPA) can be used to visualize the 3D structure of proteins at high spatial resolution (Cheng et al., 2015, Bendory et al., 2020). In contrast, by taking a series of tilted projections of the sample, cryo-electron tomography (cryo-ET) can produce tomograms depicting proteins *in situ* (McEwen et al., 2008, McIntosh et al., 2005, Doerr, 2017). Following an elaborated sequence of image analysis steps, tilt-series can be converted into tomograms and used to determine higher-resolution structures of proteins by sub-tomogram averaging (STA) (Bartesaghi et al., 2008, Bartesaghi and Subramaniam, 2009, Zhang, 2009, Wan and Briggs, 2016, Leigh et al., 2019). One essential step during the SPA and STA structure determination pipelines is the process of picking particles from 2D micrographs (Nicholson and Glaeser, 2001) or 3D tomograms (Zhang, 2009). Compared to 2D SPA, picking particles from noisy tomograms poses several additional challenges, including the higher dimensions of the data -which increases the degrees of freedom required to search for proteins in 3D-, the presence of imaging artifacts caused by the missing wedge, and the presence of confounding structural features characteristic of native *in situ* environments such as membranes, organelles and other cellular compartments.

One commonly used method for 3D particle picking is template matching (TM) (Best et al., 2007). Using an external reference that resembles the protein of interest, an exhaustive search is carried out in 3D to locate the position and orientation of proteins present within a set of tomograms. This method has two main disadvantages: 1) it requires an external template which can introduce model or reference-bias, and 2) it is computationally expensive because it requires calculation of the correlation between the template and each tomogram position for all possible orientations. Despite efforts to accelerate TM using specialized Graphical Processing Unit (GPU) hardware (Chaillet et al., 2023), the advent of high-throughput schemes for tilt-series acquisition that use beam-image shift to routinely acquire hundreds of tomograms a day (Bouvette et al., 2021, Eisenstein et al., 2023, Khavnekar et al., 2023), makes the application of TM-based particle picking approaches impractical due to their poor computational scalability.

More recently, methods based on deep learning (DL) and particularly convolutional neural networks (CNNs) have been proposed to detect particles from 2D micrographs and 3D tomograms. Examples of 2D particle picking include EPicker (Zhang et al., 2022), crYOLO (Wagner et al., 2019), Topaz (Bepler et al., 2019), and several others (Wang et al., 2016, Al-Azzawi et al., 2019, Tegunov and Cramer, 2019, Chen et al., 2017, Shiyu et al., 2022, Huang et al., 2022, Huang et al., 2021, Sanchez-Garcia et al., 2018). DL-based methods have also been used to pick particles from 3D tomograms, including approaches such as DeepFinder (Moebel et al., 2021), PickYOLO (Genthe et al., 2023) and others (Huang et al., 2022, Che et al., 2018). While these methods generally yield accurate results, they all require significant effort from users to label proteins manually necessary to train the neural networks. In addition, training the DL models is computationally expensive and requires access to specialized GPU hardware, making them incompatible with high-throughput tomography workflows. Moreover, the reliance on user-supplied labels for training can lead to bias and poor performance when these algorithms are applied to datasets that were not represented in the training set.

Here, we describe a general strategy for 3D particle picking that determines the location of particles within tomograms based solely on their expected size. Our approach is inspired by the success of algorithms used in single particle cryo-EM that detect particles based on their dimensions, such as size-based (Bartesaghi et al., 2018), Difference-of-Gaussians (DoG) (Voss et al., 2014), and Laplacian-of-Gaussians (LoG) (Zivanov et al., 2018) particle pickers. Our approach analyzes tomograms by executing the following steps: 1) building of a contamination mask in 3D to exclude regions where no particles should be present, 2) extracting candidate particle positions based on their size, and 3) filtering the set of candidate positions to eliminate false-positives. The contamination mask is used to avoid picking particles from high-contrast regions of tomograms such as areas near gold fiducials, ice contamination or other imaging artifacts. The algorithm then extracts a set of particle candidates based on their size by finding the local minima of the density within the “uncontaminated” voxels. Finally, to improve picking accuracy, each candidate position is evaluated using image-level statistics measured in local neighborhoods around each point to reduce the number of false-positives.

To test the performance of our approach we analyzed five cryo-ET datasets, four available from the Electron Microscopy Public Image Archive (EMPIAR) (Iudin et al., 2023): EMPIAR-10304 (Eisenstein et al., 2019), EMPIAR-10045 (Bharat and Scheres, 2016), EMPIAR-10064 (Khoshouei et al., 2017), and EMPIAR-10499 (Tegunov et al., 2021), and tilt-series from the dNTPase complex (Bouvette et al., 2021). These datasets represent different sample types and imaging conditions, including specimens imaged *in vitro* and *in situ*, as well as complexes ranging in molecular weight from 300 kDa to 3 MDa. We also compared the performance of our approach against a 3D extension of the fully-supervised DL-based particle picking algorithm crYOLO (Wagner et al., 2019) using two datasets of ribosomes (EMPIAR-10064 and EMPIAR-10499), and used the resulting particle coordinates to produce 3D structures by STA.

In summary, we propose a general algorithm for particle picking that accurately detects proteins from tomograms based solely on their size. Our experiments demonstrate that this approach works under a range of experimental conditions and for complexes with varying molecular weights. Bypassing the need to provide external models for template matching or extensive manual labeling needed to train fully-supervised DL-based models, our approach represents a practical and computationally efficient solution to the problem of particle picking that is compatible with modern workflows for high-throughput tomography (Liu et al., 2022).

## 2 Materials and methods

**Fig. 1:**
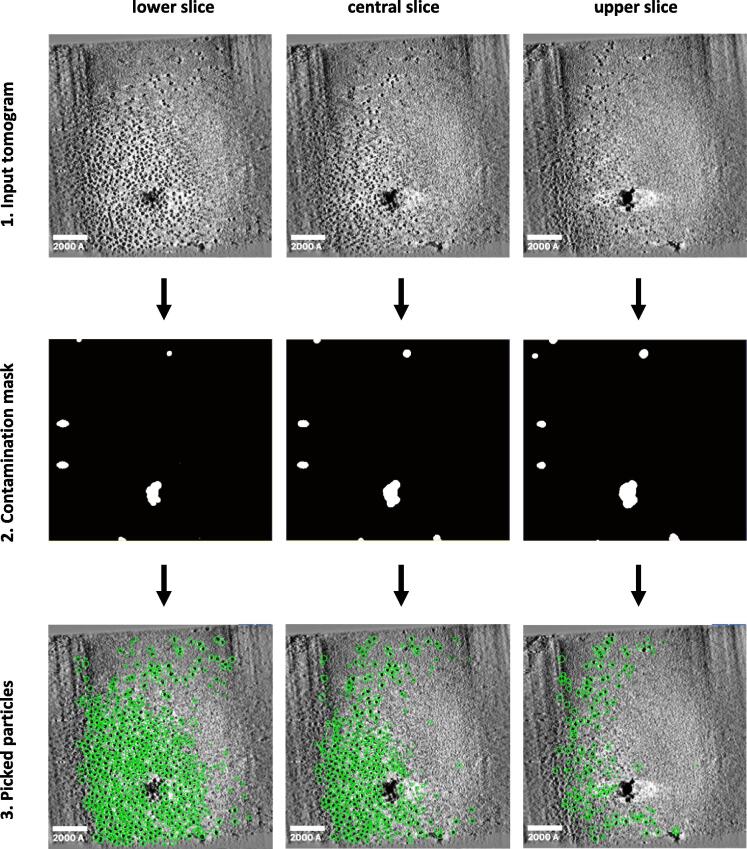
**Steps in size-based 3D particle picking strategy from cryo-ET tomograms**. Low-pass filtered tomograms are used as input (top). A binary contamination mask is then determined based on the input tomograms (middle). High-contrast features are detected automatically using robust image thresholding followed by geometric regularization to remove gaps and holes in the mask. Contamination regions are ignored for the purpose of particle picking. Particles are picked in 3D based on their dimensions (bottom, green circles). Scale bars are 2000 Å.

Tilt-series are first pre-processed following standard tomography procedures for tilt-series alignment and 3D reconstruction implemented in nextPYP (Liu et al., 2023). Our particle picking workflow is composed of three phases: 1) building of a contamination mask to eliminate undesirable regions for particle picking, 2) picking candidate particles from the contamination-free regions of each tomogram, and 3) filtering of candidate positions to eliminate false-positives, Fig. 1.

### 2.1 Contamination mask

Typical cryo-ET tomograms often contain high-contrast features that can confound particle picking algorithms, such as ice contamination, gold fiducials used as markers for alignment, hot-pixels from the detector, and other imaging artifacts. To address this problem, we propose to use a binary *contamination mask* that automatically identifs areas of the tomograms where we don’t expect to find particles useful for STA analysis.

To generate the contamination masks, we execute the following sequence of steps:

• **Gaussian filtering** – First, we apply a Gaussian filter to the input tomogram using a user-defined sigma value (200Å by default) that captures any low-frequency contrast variations and removes them from the original volume by subtraction.
• **Binary thresholding** – Second, we threshold the resulting volume using a multiple of the standard deviation of all gray values in the tomogram. Since different datasets may have voxel distributions with varying means and standard deviations, we use intrinsic image statistics to find robust thresholds that are insensitive to variations between tomograms and datasets. To allow for greater flexibility, the number of standard deviations above the mean is a user-adjustable parameter that is fixed for each dataset. After the optimal value is determined, this threshold is applied to the filtered tomograms to create the binary contamination mask.
• **Mask regularization** – The voxel-level thresholding operation done in the previous step can result in gaps, discontinuous components, or other shape irregularities. To address this problem, we apply mathematical morphology operations, including: a) cleaning (to remove connected components with sizes smaller than a user-defined threshold), b) binary closing (to remove any holes inside the contamination mask), and c) dilation (to expand the boundary of the contamination mask and avoid picking particles that are too close to the edge of artifacts).

The result of this process is a robust binary mask in 3D that is equal to 1 in areas where contamination or artifacts are present, and equal to 0 in areas of the sample where particles are located.

### 2.2 Candidate positions based on particle size

Once the contamination mask has been determined, we proceed to pick particles in areas located outside the mask. To do this, we find candidate particle positions by detecting local minima of the tomogram density within a user-specified size-range. We first apply a low-pass filter to the original tomogram, which improves image contrast by removing high-resolution noise and making features in the expected size-range more prominent. Assuming that the center of each particle is darker than the surrounding density, we find candidate particle positions by detecting local minima in the filtered 3D tomograms. Local minima are determined using cubic neighborhoods of size $h^{3}$. Assuming that the minimum distance between neighboring particle centers is 2 times the particle radius *r*, we set h=4r to guarantee that neighboring particles do not overlap with each other. After ignoring all local minima that fall inside the contamination mask, we have a set of candidate positions corresponding to individual particle locations.

While using a neighborhood size of h=4r can usually find most particle candidates, it may also produce false-negatives. For example, if one point is a particle center, the value at that point should be a local minima within a distance of *r* to the center and there should be no other particle centers within a surrounding area of radius 2r. However, there may be voxels within a 2r radius that have smaller gray values than the center which are not inside the particle and may not be centers of other particles, and this can lead to many false-negatives. To solve this problem, we use a smaller value of h=2r together with an iterative strategy to eliminate false particle positions, Supp. Fig. 1. While this strategy takes longer to run and may occasionally introduce some false-positives, overall, it is an effective method to increase the number of true-positives.

### 2.3 Filtering particle candidates

The final step in our workflow consists in filtering the candidate particle positions to remove any remaining false-positives. For each position determined in the previous step, we extract a sub-tomogram of size ${\left(3r\right)}^{3}$ centered at each selected position and calculate its 2D projection along the Z-axis. We use 2D projections to reduce the dimensionality of the data and improve computational efficiency. A central circle with size equal to the particle radius is considered as the *foreground* region, and we calculate the standard deviation of gray values within each projection to decide whether the position is a true particle. If the standard deviation of the foreground is above a given threshold, the position is considered as a true particle. Similar to the binarization threshold used to determine the contamination mask, the threshold here is calculated with respect to the mean density plus a multiple of the standard deviation of gray values within each projection. In this case, the number of standard deviations above the mean is also a user-adjustable parameter.

For *in situ* datasets that contain strong features such as membranes or other cellular compartments, this procedure can result in many false-positives. To deal with these situations, we introduce an additional criteria that only considers a point as a true-positive if the standard deviation of the *background* (the complement of the foreground for a given candidate particle) is smaller than the standard deviation of the *foreground*. By applying this criterion, we successfully eliminate particle positions in areas where the standard deviation of the background is high due to the presence of high-contrast objects.

In addition to the shape-based contamination mask and particle filtering steps, we also restrict particle locations to be within a certain distance from the middle plane of the tomogram. This step is included to account for the finite thickness of the slab containing the sample, and it helps eliminate additional false-positives. This parameter is set to a large value by default, ensuring that candidate particles from all slices in a tomogram will be selected.

## 3 Results

To validate the performance of our approach, we analyzed five sets of tilt-series obtained from *in vitro* and *in situ* samples and evaluated the particle picking results both qualitatively and quantitatively. For two of the datasets, we compared the performance of our approach against a 3D extension of the deep-learning based algorithm crYOLO (Wagner et al., 2019). We also used the particle picking positions resulting from each algorithm to generate 3D reconstructions using STA and compared the map resolutions obtained in each case. Table 1 shows a summary of the configurations and parameters used for the five datasets and corresponding quantitative results. The average processing times per tomogram are shown to illustrate the computational efficiency of our size-based particle picking approach.

**Table 1:** **Acquisition, data processing parameters, and picking results for the five datasets processed by our size-based approach.** In all cases, tilt-series were pre-processed and aligned using nextPYP (Liu et al., 2023), and tomograms were reconstructed with dimensions of 512 × 512 × 256 voxels.

Dataset	EMPIAR-10304	EMPIAR-10045	EMPIAR-10064	EMPIAR-10499	dNTPase
*Imaging conditions*					
Scope voltage (kV)	300	300	300	300	300
Tilt-range	±60	±60	±60	±60	±36
Pixel size (Å)	2.10	2.17	2.62	1.70	1.35
Particle radius (Å)	100	90	70	90	55
Number of tilt-series	12	7	4	64	205
					
*Picking parameters*					
Threshold (for contamination)	2.5	1.5	2	0.75	1
Minimum size (for contamination)	125	125	125	125	125
Dilation size (for contamination)	70	70	100	70	70
Detection size	128	128	128	128	32
Threshold (for final filtering)	1.2	1.5	2	0.3	−0.1
					
*Results*					
Particles detected	12,796	3,759	7,897	30,285	133,354
Running time per tomogram (seconds)	78.3	101.5	127.1	118.9	142.8

### 3.1 Detection of ribosomes from *in vitro* samples

**Fig. 2:**
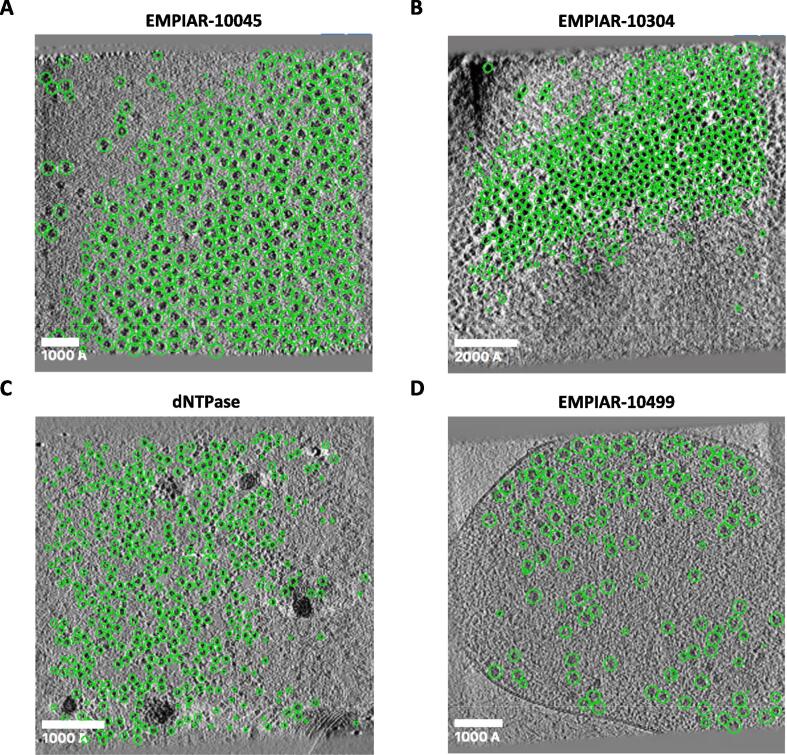
**Validation of size-based particle picking using*****in vitro*****and*****in situ*****cryo-ET datasets.** Results of size-based particle picking on four cryo-ET datasets. 2D slices of representative tomograms obtained from four datasets of samples with molecular weights ranging between 300 kDa and 3 MDa. Selected particles obtained using the size-based approach are shown as green circles on tomograms from EMPIAR-10045 (**A**), EMPIAR-10304 (**B**), dNTPase (**C**), and EMPIAR-10499 (**D**). The contamination masks help prevent picking particles from high-contrast areas corresponding to ice contamination, cell membranes, etc. Scale bars are 1000 Å for EMPIAR-10045, dNTPase, and EMPIAR-10499, and 2000 Å for EMPIAR-10304.

We first applied our approach to tilt-series from EMPIAR-10045 (Bharat and Scheres, 2016), EMPIAR-10064 (Khoshouei et al., 2017), and EMPIAR-10304 (Eisenstein et al., 2019). These *in vitro* datasets of purified ribosomes only have a few obvious contamination areas and artifacts which our approach was able to easily avoid. Due to the large molecular weight of ribosomes and the high defocus used for imaging, particles in these datasets have high contrast making them easy to detect by our approach, Fig. 2A, B and Supp. Fig. 2.

### 3.2 Detection of lower molecular weight particles

While ribosomes play an integral role in cell biology, the vast majority of molecules encoded in the genome have molecular weights of 300 kDa or less. Proteins in this family are difficult to detect in tomograms due to their lower scattering masses that produce weaker contrast. To evaluate the ability of our approach to detect these targets, we analyzed tomograms of a monodisperse sample of protein VC1979 of *Vibrio cholerae* (dNTPase), an HD-domain family protein and a homolog of SAMHD1 and *E. coli* dGTP triphosphohydrolase (Bouvette et al., 2021). We analyzed 205 tomograms and successfully detected 49,400 particles which closely matched the manually picked positions reported in the original study (Bouvette et al., 2021), Fig. 2C and Supp. Fig. 3. Given that this sample was frozen into a very thin layer of ice, we limited the detection to 32 slices around the central plane to reduce the number of false-positives. The use of the contamination mask also significantly reduced the number of false positives in this dataset, producing accurate picking results despite the lower molecular weight of the complex.

### 3.3 Particle detection from crowded cellular tomograms

The ultimate goal of cryo-ET/STA is the determination of high-resolution structures imaged inside cells. To assess the ability of our approach to pick particles from crowded cellular environments, we analyzed tilt-series from native *M. pneumoniae* cells treated with chloramphenicol available from EMPIAR-10499 (Tegunov et al., 2021). Compared to *in vitro* samples, cellular datasets pose additional challenges for particle picking because tomograms may include cell membranes and other high-contrast features that can confuse particle picking algorithms. Use of the smaller neighborhood size of h=2r was important to detect many particles that were missed by the method that used h=4r. The filtering step where positions are eliminated according to the local image statistics was also critical to remove candidate particles at the location of cell membranes and edges, Fig. 2D.

### 3.4 Detection accuracy compared to crYOLO

**Fig. 3:**
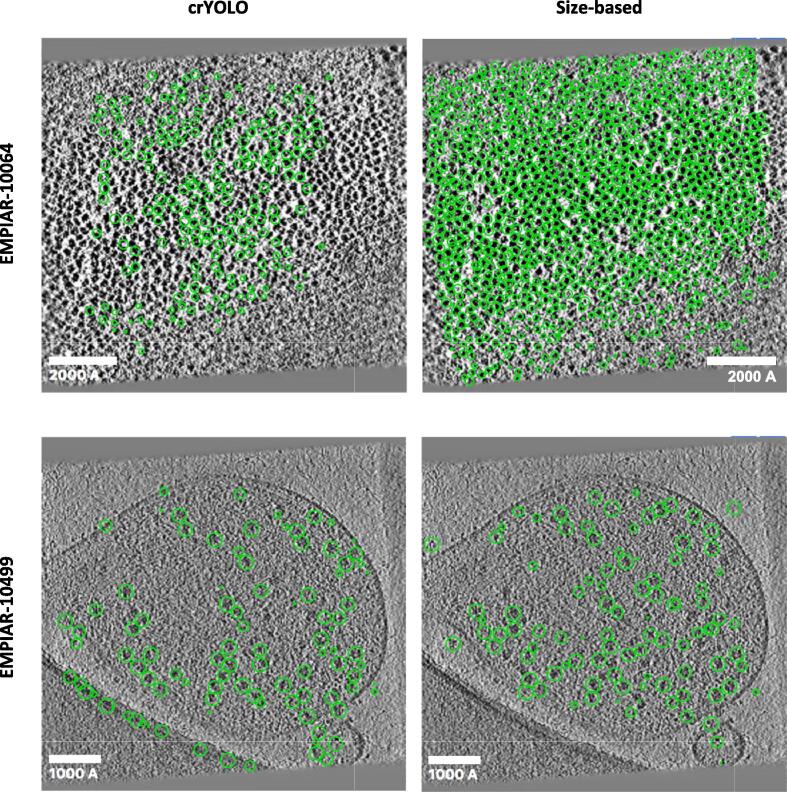
**Comparison between size-based and crYOLO particle picking**. Representative slices of tomograms showing particles picked using crYOLO and size-based particle picking from EMPIAR-10064 and EMPIAR-10499. crYOLO was trained on each dataset using manually selected labels from multiple consecutive slices. Representative tomogram of 70S ribosomes from EMPIAR-10064 with particles selected by crYOLO and our size-based approach (top). Representative tomogram of 80S ribosomes inside cells from EMPIAR-10499 with particles selected by crYOLO and our size-based approach (bottom). crYOLO produces many false-positives along the edge of the carbon and cell membranes that are avoided by our size-based approach. Scale bars are 2000 Å for EMPIAR-10304, and 1000 Å for EMPIAR-10499.

Next, we compared the performance of our approach against a 3D extension of the original crYOLO algorithm (Wagner et al., 2019) (https:cryolo.readthedocs.io) using tomograms of ribosomes imaged *in vitro* (EMPIAR-10064) and *in situ* (EMPIAR-10499). Since crYOLO is a fully-supervised deep learning algorithm, we manually annotated four tomograms per dataset to use as the training set. Annotation in crYOLO is very time consuming as it requires labeling each particle in multiple slices. For training the models, we manually labeled 2,576 particles from a single tomogram from EMPIAR-10064, and 1,226 particles from two tomograms from EMPIAR-10499. We then compared the algorithms in terms of numbers of particles detected and running time (Table 2), as well as precision, recall, and F1 scores (Table 3). For the *in vitro* 80S ribosome dataset (EMPIAR-10064), we detected many more particles than crYOLO. For the *in situ* ribosome dataset (EMPIAR-10499), crYOLO produced many false-positives along high-contrast areas such as edges and cell membranes while our approach managed to avoid them. In terms of running time, while the inference stage for crYOLO runs fairly quickly, training is very time consuming and resulted in significantly lower computational efficiency compared to our approach. The qualitative results of the comparison are shown in Fig. 3.

**Table 2:** **Comparison of results between our size-based approach and crYOLO.** We processed datasets EMPIAR-10064 and EMPIAR-10499 using both approaches and compared the picking results and running time statistics. In terms of accuracy, our size-based approach picks more particles (especially in EMPIAR-10064). The inference stage in crYOLO is quite fast, but it requires long training times which is not required in our size-based approach.

Dataset	EMPIAR-10064	EMPIAR-10499
*Size-based results (running on Intel Xeon Gold 6154 CPU @ 3.00 GHz)*		
Total particles detected	7,897	30,285
Time for initialization (seconds)	3.3	3.2
Time for contamination detection (seconds)	37.7	61.4
Time for particle detection (seconds)	86.1	54.3
Total runtime per tomogram (seconds)	127.1	118.9

*crYOLO results (running on one NVIDIA V100 GPU with 32* *GB of RAM)*		
Total particles detected	1,593	27,896
Inference time per tomogram (seconds)	32	33
Training time per annotated tomogram (seconds)	5,199	4,238
Total training time for 4 annotated tomograms (seconds)	20,796	16,952

**Table 3:** **Quantitative comparison of results between our size-based approach and crYOLO.** Using manually picked positions as ground-truth, we calculated precision, recall, and F1 scores on tilt-series from EMPIAR-10064 and EMPIAR-10499 for both picking methods. For EMPIAR-10064, crYOLO's higher rate of false positives resulted in lower recall and F1 scores compared to size-based picking. For EMPIAR-10499 both approaches picked a similar number of particles, but the size-based approach produced higher precision, recall, and F1 scores (highest values for each dataset are underlined).

Dataset	EMPIAR-10064		EMPIAR-10499	
	*Size-based*	*crYOLO*	*Size-based*	*crYOLO*
Precision	0.578	0.968	0.878	0.855
Recall	0.961	0.122	0.816	0.616
F1 score	0.722	0.216	0.846	0.716

### 3.5 Improved picking performance leads to higher-resolution structures

**Fig. 4:**
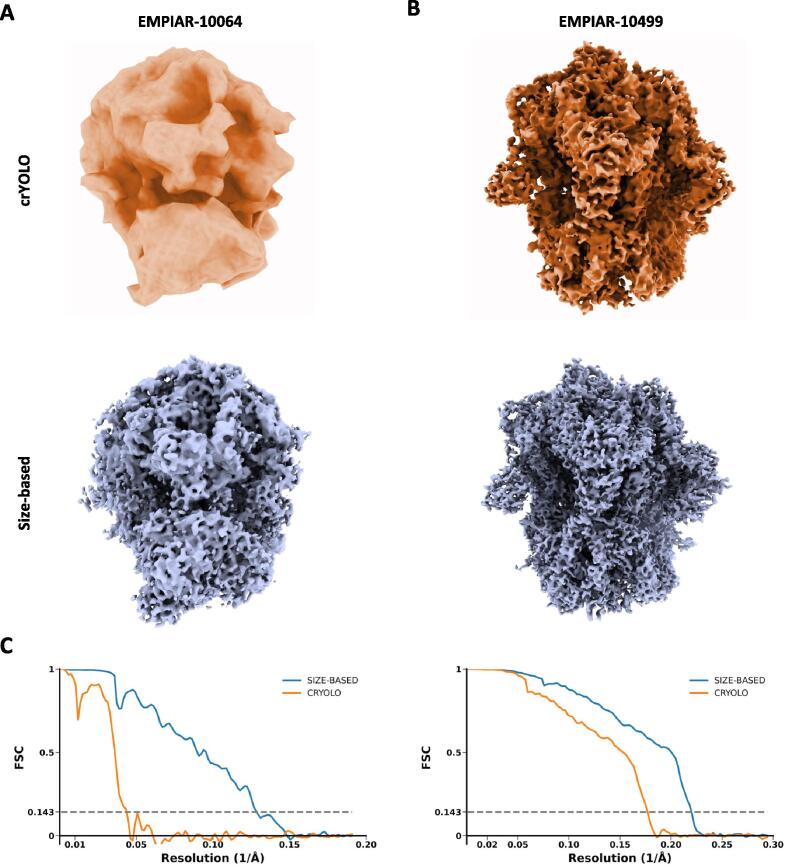
**Maps from particles obtained using our size-based approach and crYOLO.** Reconstructions obtained by sub-tomogram averaging from particles produced using our size-based particle picking approach and crYOLO for tomograms from EMPIAR-10064 and EMPIAR-10499. **A**. For EMPIAR-10064, crYOLO only selected 1,593 particles resulting in a significantly lower resolution map (23.0 Å) while our size-based approach produced 7,897 particles leading to a 7.8 Å resolution map. **B**. For the *in situ* dataset EMPIAR-10499, crYOLO produced 27,896 particles and produced a map at a lower resolution of 5.7 Å, while the size-based approach produced 30,285 particles leading to a 4.5 Å resolution map. **C**. Corresponding Fourier Shell Correlation (FSC) curves between half-maps are shown in both cases indicating the estimated resolution using the 0.143-FSC cutoff criteria. Other data processing details and statistics are included in Table 3.

Lastly, we used particles picked by crYOLO and our approach to produce 3D reconstructions using STA from EMPIAR-10064 and EMPIAR-10499. After obtaining the particle positions, we subjected each set of particles to STA analysis as implemented in nextPYP. In both cases, the final maps obtained from particles selected using our approach had higher resolution, both due to the lower false-positive rate and the higher number of particles, Fig. 4. Data processing statistics are included in Table 4.

**Table 4:** **STA data processing statistics for EMPIAR-10064 and EMPIAR-10499.** Number of particles picked, used for reconstruction, and final map resolutions are reported for crYOLO and our size-based approach.

Dataset	EMPIAR-10064		EMPIAR-10499	
	Size-based	crYOLO	Size-based	crYOLO
Total particles picked	7,897	1,593	30,285	27,896
Particles used (after cleaning)	3,756	1,593	24,885	13,495
Resolution (Å)	7.8	23.0	4.5	5.7

## 4 Discussion

Recent advances in cellular sample preparation (Klumpe, 2021, Kelley et al., 2022), specimen screening (Bouvette et al., 2022) and high-speed tomography (Bouvette et al., 2021, Eisenstein et al., 2023, Khavnekar et al., 2023), have effectively increased the throughput of tomography data collection making it possible to acquire hundreds of tilt-series a day (Liu et al., 2022). Development of effective strategies for cryo-ET/STA data analysis such as particle picking, however, are still lacking and require extensive user input and access to specialized compute resources, constituting an important bottleneck.

Based on the success of 2D blob-based strategies used in cryo-EM/SPA, we propose a 3D size-based particle picking algorithm that efficiently locates proteins within tomograms without requiring external templates or labeled data for training. By avoiding areas of contamination and effectively reducing the number of false-positives, our approach achieves state-of-the-art results and avoids picking particles from cell membranes and edges. Unlike existing methods based on template-matching and deep learning, our approach is computationally efficient because it does not require exhaustive orientation search, manual labeling, or training of neural networks. When compared with fully-supervised deep learning methods like crYOLO, our approach not only results in higher precision, recall and F1 values, but also runs faster and does not require hardware accelerators.

Our approach also has some limitations. First, since we do not explicit account for the missing wedge, the results can be biased by imaging artifacts resulting from the limited tilt range. Second, the method may struggle to detect particles from specific *in situ* samples such as lattice or microtubule-attached targets, as well as membrane proteins or COPI/COPII particles due to the strong background. However, our algorithm does work on hollow or ”doughnut” shapes provided that the value of the low-pass filter is set correctly.

Our size-based particle picking approach can be conveniently accessed using nextPYP’s graphical user interface (GUI). Once tomograms have been reconstructed, users must select ”auto” as the particle detection method. To choose the particle radius for detection, users can simply measure the size of particles directly on the tomograms using nextPYP’s interactive measuring tool. Immediately after execution, detected particle coordinates are visualized on the original tomograms giving users real-time feedback on the accuracy of particle detection.

In summary, we propose a size-based method for 3D particle picking that enables accurate and computationally efficient particle picking from tomograms representing a range of samples. With proper parameter tuning, the algorithm can be optimized to work on heterogeneous datasets, including complexes with varying molecular weight and imaged both *in vitro* and *in situ*. Our algorithm produces accurate particle positions with low false-positive rates that can be used to determine high-resolution structures by STA. Unlike template-matching and DL-based approaches, our approach does not require the use of external references or manual labeling of particles, and does not need specialized GPU hardware to run, thus allowing the routine processing of datasets with hundreds of tomograms. In general, while the effectiveness of deep learning-based and template-search methods has been previously demonstrated, the addition of our size-based approach to the battery of methods for particle picking represents a valuable alternative for cryo-ET practitioners.

## Code availability

The source code is available at: https://gitlab.cs.duke.edu/bartesaghilab/cet_pick_size and the algorithm has been incorporated into the software package nextPYP ( https://nextpyp.app).

## Declaration of competing interest

The authors declare the following financial interests/personal relationships which may be considered as potential competing interests: Alberto Bartesaghi reports financial support was provided by The Chan Zuckerberg Initiative. Alberto Bartesaghi reports financial support was provided by National Institute of General Medical Sciences. Alberto Bartesaghi reports financial support was provided by National Institute of Allergy and Infectious Diseases. If there are other authors, they declare that they have no known competing financial interests or personal relationships that could have appeared to influence the work reported in this paper.

## Data Availability

The data used for this study was downloaded from the EMPIAR database.
