# Supplementary material for: Accurate size-based protein localization from cryo-ET tomograms
Source: J Struct Biol X. 2024 Jun 26;10:100104. doi: 10.1016/j.yjsbx.2024.100104 (PMC11263962; doi:10.1016/j.yjsbx.2024.100104)
Supplement: Supplementary data 1 [file mmc1.pdf]

ARTICLE

Accurate size-based protein localization from cryo-ET tomograms

Weisheng Jin,[1] Ye Zhou,[1] and Alberto Bartesaghi[*,1,2,3]

[1]Department of Computer Science, Duke University, Durham, USA
[2]Department of Biochemistry, Duke University School of Medicine, Durham, USA
[3]Department of Electrical and Computer Engineering, Pratt School of Engineering, Duke University, Durham, USA

*Corresponding author: alberto.bartesaghi@duke.edu

Supplementary Figures 1-3

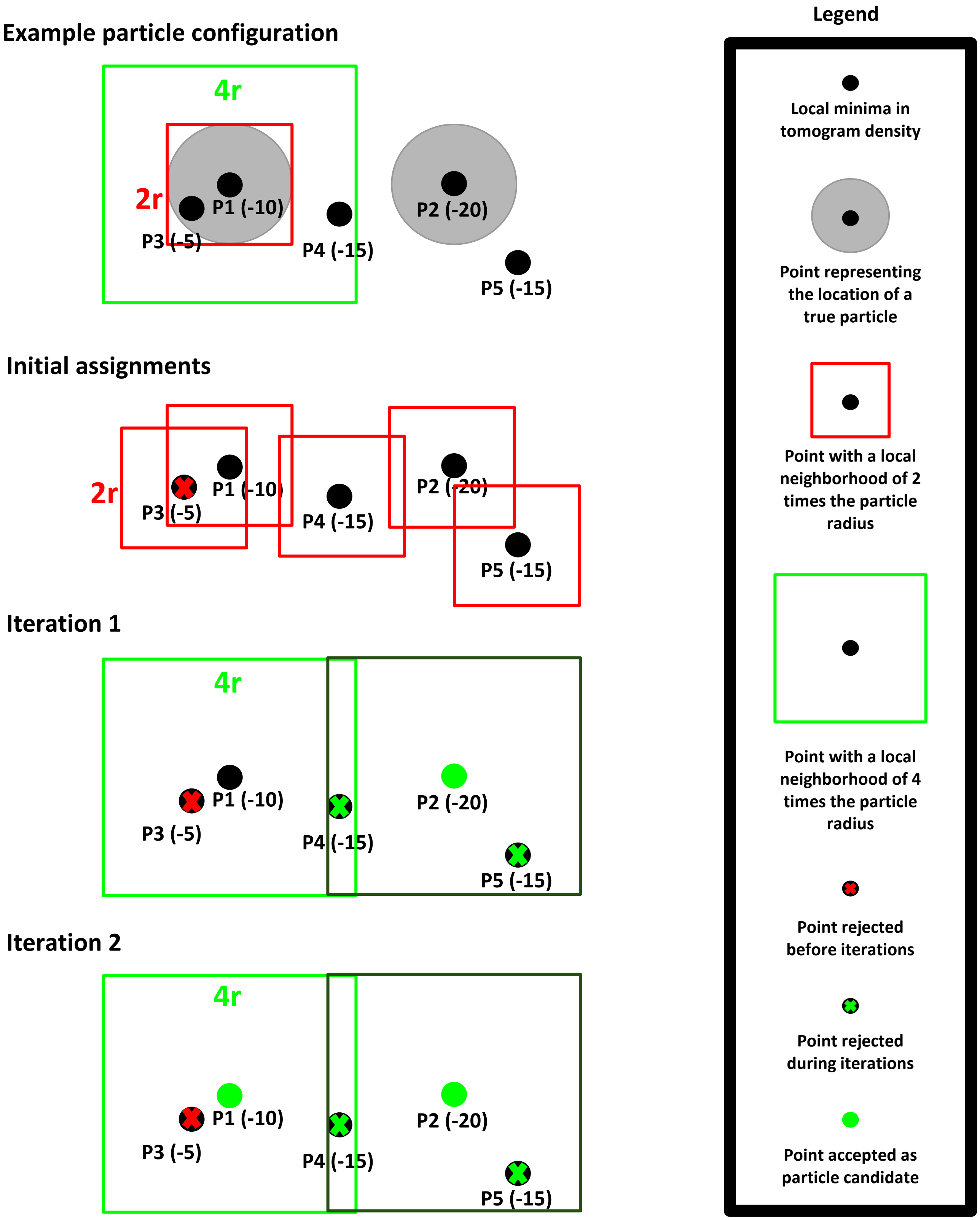

Supplementary Figure 1 | Iterative strategy for improving particle picking accuracy.
Example configuration of local minima corresponding to particle locations within a tomogram. Five points (represented as dark circles) represent local minima of the density and are considered as potential candidates to be particles. Below each circle, we show the identity of each point (P1 to P5) and their grey values. The two solid grey circles are centered around two true particles. The red square of size 2 times the particle radius (2r) and a green square with size 4 times the radius (4r) mark the surrounding areas of each point. P3 is first discarded as a candidate particle (red cross) during the initial assignments because there's a darker point in its surrounding area of side length 2r. In the first iteration, P2 is chosen as a candidate particle center (becomes green) while P4 and P5 are discarded (green crosses). In the second iteration, P1 is chosen to be a candidate particle (becomes green), resulting in the successful detection of true particles at points P1 and P2.

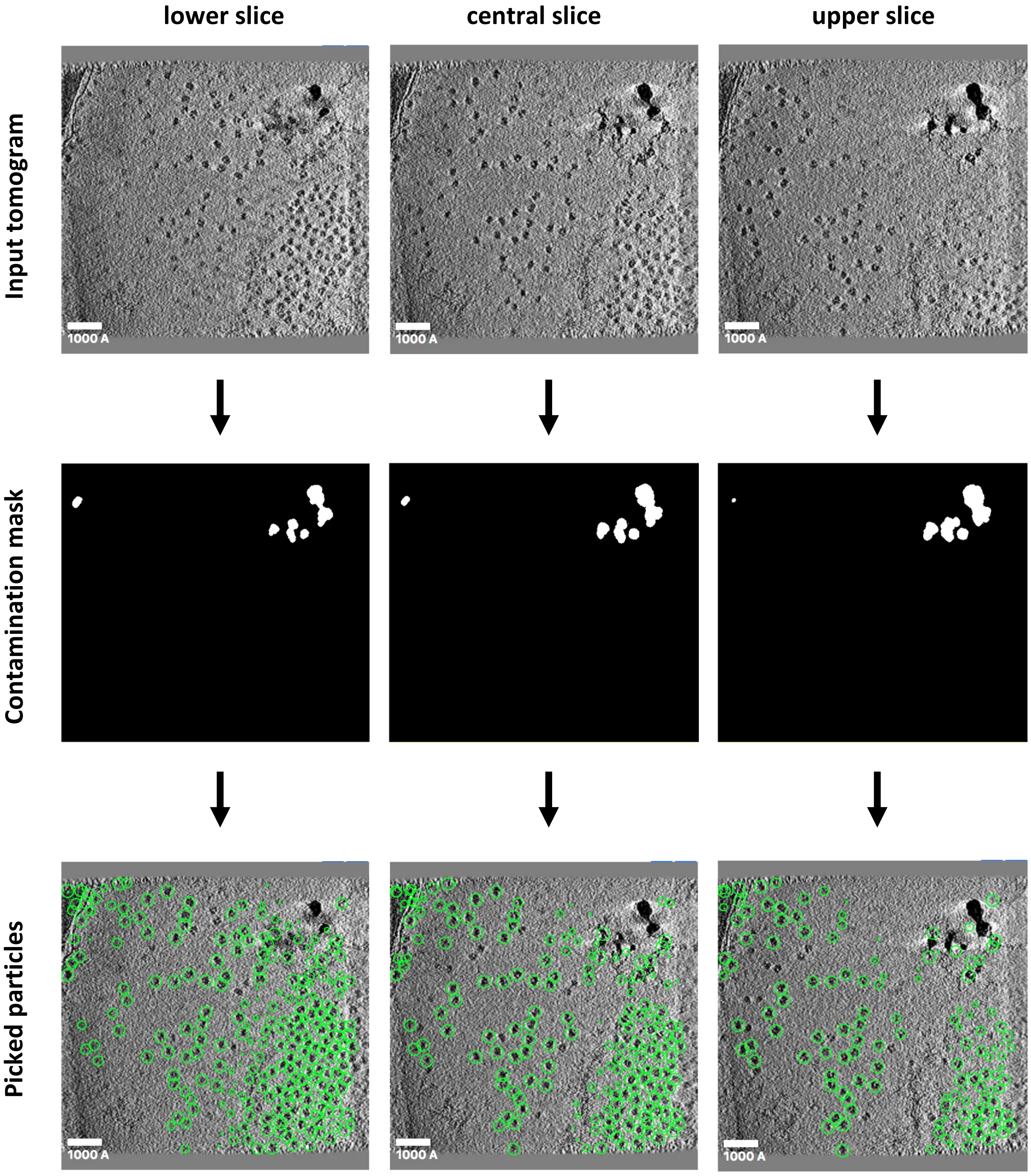

Supplementary Figure 2 | Particle picking steps on a tilt-series from EMPIAR-10045.
Consecutive slices from a representative tomogram from EMPIAR-10045 containing purified S. cerevisiae 80S ribosomes are shown (top), followed by the results of the contamination detection step (middle). Final particle locations are detected based on the expected size of the ribosomal complexes and from areas that are not labeled as contamination (bottom). Scale bars are 1000 Å.

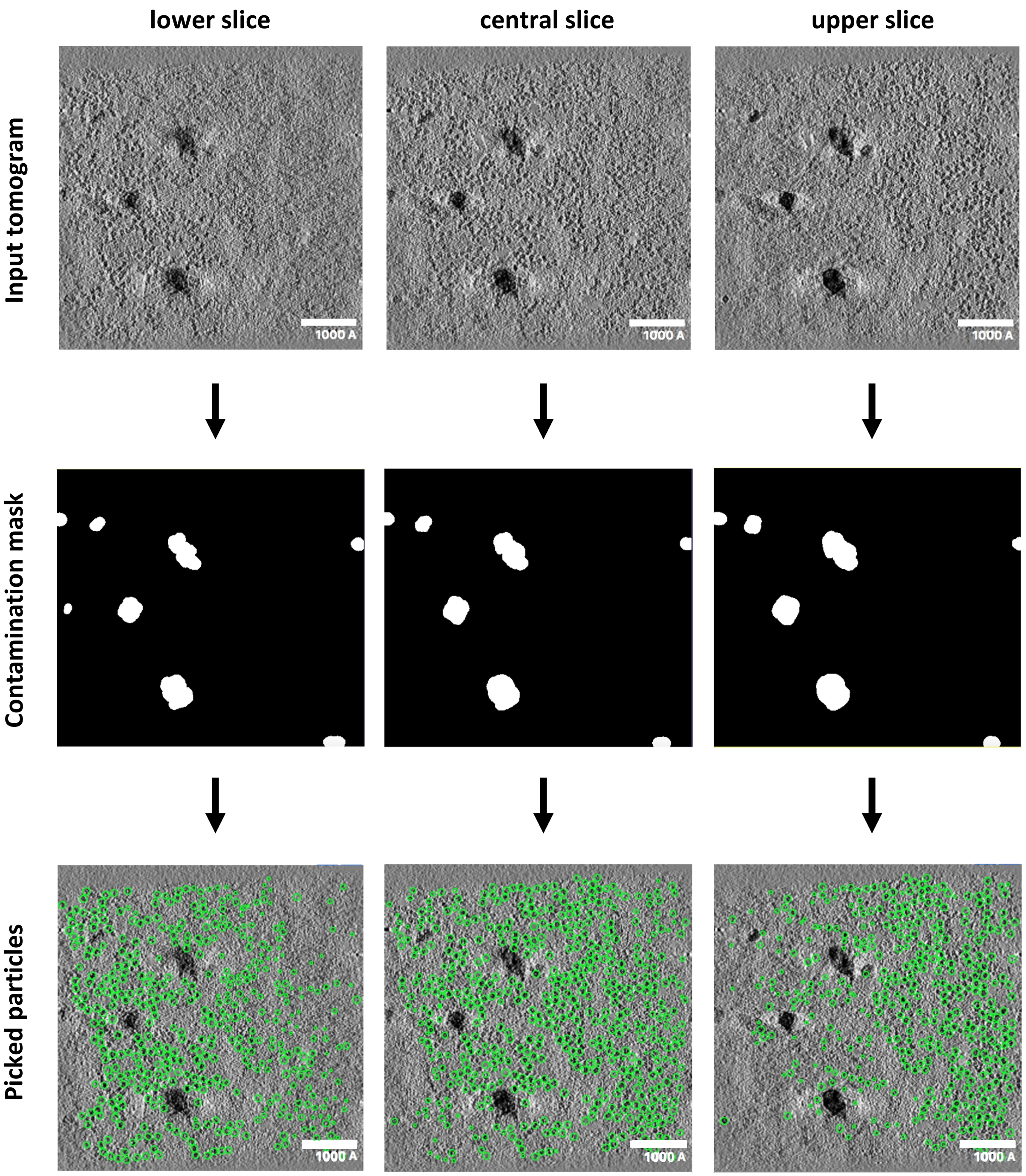

Supplementary Figure 3 | Size-based particle picking of a dNTPase (300kDa) sample.
Consecutive slices from a representative tomogram from a *in vitro* sample of dNTPase (300kDa) are shown (top), followed by the results of the contamination detection step (middle). Final particle locations are detected based on the expected size of the complex (75 Å) and from areas that are not labeled as contamination (bottom). Scale bars are 1000 Å.
